# Detection of *Brucellae* in peripheral blood mononuclear cells for monitoring therapeutic efficacy of brucellosis infection

**DOI:** 10.1186/s13756-019-0607-2

**Published:** 2019-10-15

**Authors:** Heng Yang, Guoxia Zhang, Peifang Luo, Zuoping He, Feihuan Hu, Linhai Li, Jean-Pierre Allain, Chengyao Li, Wenjing Wang

**Affiliations:** 1 Department of Transfusion Medicine, School of Laboratory Medicine and Biotechnology, Southern Medical University, Guangzhou, 510515 China; 2 Department of Infectious Disease, Hei Longjiang General Hospital of Agriculture Reclamation Bureau, Harbin, 150088 China; 3 The Sixth Affiliated Hospital of Guangzhou Medical University, Qingyuan People’s Hospital, Qingyuan, 511500 China; 4 The Fifth Affiliated Hospital of Sun Yat-sen University, Zhuhai, 519000 China; 5 Department of Blood Transfusion, Guangdong Provincial People’s Hospital, Guangzhou, 510120 China; 6 Department of Laboratory Medicine, General Hospital of Southern Theatre Command of PLA, Guangzhou, 510010 China; 7 Emeritus professor of Transfusion Medicine, University of Cambridge, Cambridge, CB2 2PT UK

**Keywords:** Brucellosis, Treatment, *Brucella* diagnosis, PBMC

## Abstract

**Background:**

Brucellosis is one of the most severe widespread zoonoses caused by the Gram-negative bacterium *Brucella species*. The diagnosis and clinical assessment of human brucellosis are very important for the management of patients, while there is a lack of effective methods to detect *Brucellae*. Classical culture of *Brucella* species is time consuming and often fails. A simple and sensitive assay is needed for diagnosis of *Brucella* infection and monitoring of treatment in man.

**Methods:**

Blood samples and peripheral blood mononuclear cells (PBMCs) were collected from 154 patients hospitalized for brucellosis. *Brucella* antibodies were detected by Rose Bengal Plate Test (RBPT), Standard Tube Agglutination Test (SAT) and enzyme-linked immunosorbent assay (ELISA). Intracellular *Brucellae* were detected by blood culture and immunofluorescence staining (IFS).

**Results:**

Among 154 brucellosis patients, 59.7% (92/154) were antibody reactive by RBPT, 81.8% (126/154) by SAT and 95.5% (147/154) by ELISA, respectively. Only 3.2% (5/154) of patient blood samples resulted in positive *Brucella* culture, while 68.8% (106/154) carried IFS detectable *Brucella* antigens in PBMCs. Gender (*P* = 0.01) but not age (*P* > 0.05) was a significant risk factor. The frequency of intracellular *Brucella* antigens was similar between patients receiving different treatment regimens (*P* > 0.05). However, a significant decrease of intracellular *Brucellae* was observed only in patients with acute brucellosis after the third course of treatment (*P* < 0.05), suggesting that current regimens to treat chronic brucellosis were not effective.

**Conclusions:**

IFS appears a sensitive assay for detection of *Brucella* antigens in PBMCs and could be used for diagnosis and therapeutic monitoring of brucellosis in clinical practice.

## Introduction

Brucellosis is one of the most severe widespread zoonoses in the developing world and is caused by the Gram-negative bacterium *Brucella species* [1]. Intracellular *Brucella spp.* is often detected in chronic disease, and usually persists lifelong [2]. Clinical manifestations of human brucellosis include fever, profuse sweating, joint and muscle pain, hepatomegaly and splenomegaly, osteomyelitis, arthritis and sacroiliitis, etc., severely impacting patient’s quality of life [3–5]. Early diagnosis and treatment of brucellosis could significantly improve patient prognosis. Isolation of the organism from cultured blood samples was the diagnostic gold standard. Meanwhile serological tests were used to diagnose human brucellosis together with patient’s clinical and epidemiological history. Culture requires 3–5 days to develop visible colonies, but *Brucella spp.* grows slowly, so it may take as long as over 2 weeks to obtain a definitive result. Due to its pathogenicity, a biosafety level 3 laboratory (BSL-3) is mandatory when handling *Brucellae* [6]. A faster and safer brucellosis laboratory testing method should be established, especially in developing countries. In this study, previously developed immunofluorescence cell staining (IFS) was utilized to detect intracellular bacteria [7, 8] and was applied for diagnosis and monitoring of patients infected with *Brucellae*.

## Materials and methods

### Blood samples

A number of 154 blood samples were collected from brucellosis patients at the General Hospital of Agricultural Reclamation Bureau, Harbin, Heilongjiang, China. All patients were initially diagnosed with brucellosis by clinical examination in combination with serological testing and bacterial culture, including the Rose Bengal Plate Test (RBPT), the Standard Tube Agglutination Test (SAT) and blood culture. Peripheral blood mononuclear cells (PBMCs) were prepared for detection of intracellular *Brucellae* according to the manufacturer’s instructions (Ficoll Pague PLUS, GE Healthcare Life Sciences).

The control blood samples were collected in Guangzhou blood center, Guangdong province where brucellosis is non-endemic. Blood donors passed the predonation questionnaire, including lack of fever but no question addressed brucellosis history. The blood samples were routinely screened with two different enzyme immunoassays for HBsAg and antibodies to HCV, HIV-1/2, and syphilis [9, 10]. Thirty-six blood donors with negative serologic tests and normal ALT level were selected to test for *Brucella* infection.

### Immunofluorescence staining (IFS) of PBMCs

Intracellular *Brucellae* in patient’s PBMCs were detected by IFS [7]. PBMCs were isolated from 3 ml of fresh EDTA venous blood by Ficoll Hypaque, then transferred in a culture plate for 2 h in order to let cells attach. Secondly, cells attached on the plate were fixed and individually incubated with a monoclonal antibody (mAb) as primary antibody, such as mAb 2C1, 5H3, 2A4 or 5A5 against Bp26 or Omp31 protein of *Brucellae* [7, 8]. MAb 2E12 to HCV NS3 was used as negative control [11]. Alexa Fluor 594-conjugated goat anti-mouse secondary IgG (H + L) (Invitrogen China Limited, Guangzhou, China) or Alexa Fluor 594-conjugated Affinipure Goat Anti-Mouse IgG + IgM (H + L) (Jackson ImmunoResearch Laboratories, Inc., USA) were used as secondary antibody. The stained cells were examined by a NikonLabophot photomicroscope with the epifluorescence attachment EF-D (Nikon, Garden City, NY, USA).

### *Brucella* blood culture

Five to 10 ml of peripheral blood were cultured for *Brucellae* using an automatic blood culture system (Biomerieux Co. Ltd., Bact/ALERT 3D 60, Lyon, France) with an average culture time of 5–7 days, as previously described [12]. Visible bacteria colonies were identified using automatic microbial identification machine (Biomerieux Co. Ltd., VITEK 2 COMPACT 30).

### Serologic assays

Patients’ sera were retrospectively re-tested by RBPT and SAT according to the manufacturer’s instructions (Biovaccine Co., Ltd., Harbin Pharmaceutical Group, Harbin, China). Antibody titer of patient’s sera tested with SAT equal to or over 1:100 indicated a diagnosis of Brucellosis in addition to chronic patients with epidemiological exposure history carrying low titer antibody such as 1:50. Sera were also tested with an enzyme-linked immunosorbent assay (ELISA) (*Brucella* IgG ELISA Kit, Neobioscience Technology CO., LTD).

### Treatment of human brucellosis

Brucellosis patients were treated with a combination of at least two compatible drugs according to presentation and condition. Intravenous treatment included Etimicin (100 mg ivgtt, twice), Enoxacin (0.2 g ivgtt, twice), Levofloxacin (0.6 g ivgtt, once), Ceftazidime (3 g ivgtt, twice) or Cefperazone-Sulbactam (3 g ivgtt, twice), together with a drug taken orally, including Rifampicin (0.45–0.6 g p.o., once in the morning) or Doxycycline (0.1 g p.o., twice). Some volunteer patients already on combined drug treatment additionally received immune enhancing drugs such as Thymopeptides and Placental Polypeptides (Guizhou Taibang Biological Products Co., LTD., Chinese Food and Drug Administration (CFDA) approval number: H20046260). Acute brucellosis was treated in hospital with multiple standard 20 days courses of treatment separated by an interval of 10 days.

### Statistical analysis

Computer software (SPSS, Version 21.0, SPSS, Inc., Chicago, IL) was used for statistical analysis. Experimental data was analyzed by chi-square test and T test. Abnormal distribution data was analyzed by multiple-sample nonparametric test. A *P* value of less than 0.05 was considered significant.

## Results

### Characterization of patients with *Brucella* infection

**Fig. 1:**
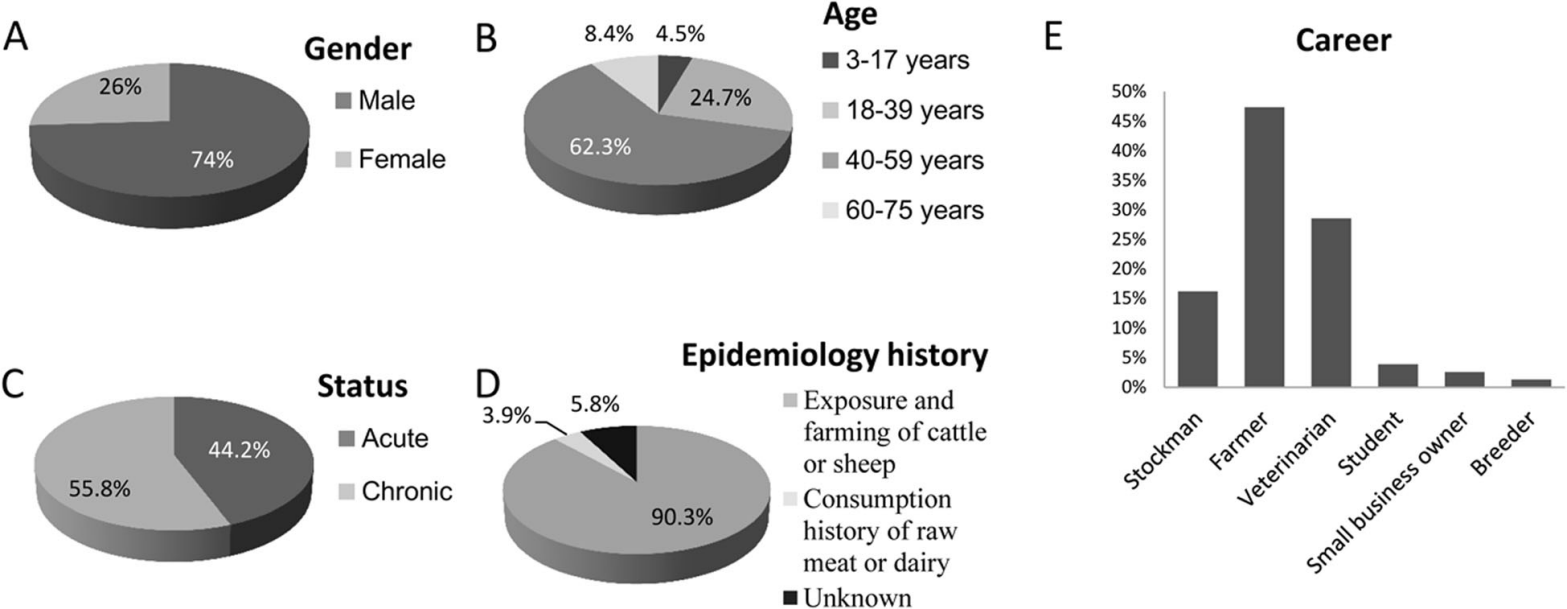
Baseline demographics of patients with brucellosis. **a-e** Brucellosis patients were classified by their gender, age, disease status, epidemiology history and occupation, respectively

A total of 154 patients were enrolled in this study (Additional file 1: Table S1). Males were 114 (74.0%) and 40 (26.0%) were female, ranging in age between 3 to 73 years with a mean age of 43.4 ± 13.5 years. Patients were stratified in two clinical groups: acute phase (with symptoms for less than 6 months) and chronic phase (with symptoms for more than 6 months). There were 68 acute and 86 chronic cases, respectively. The epidemiologic investigations indicated that 139 patients (90.3%) experienced close contact with animals, six patients (3.90%) had consumed raw meat or dairy products, and the remaining nine patients did not report at risk circumstances. Occupations were 25 (16.20%) livestock keepers, 73 (47.40%) farmers, 44 (28.57%) veterinarians, 6 (3.9%) students, 4 (2.60%) small business owners and 2 (1.30%) breeders (Fig. 1).

**Fig. 2:**
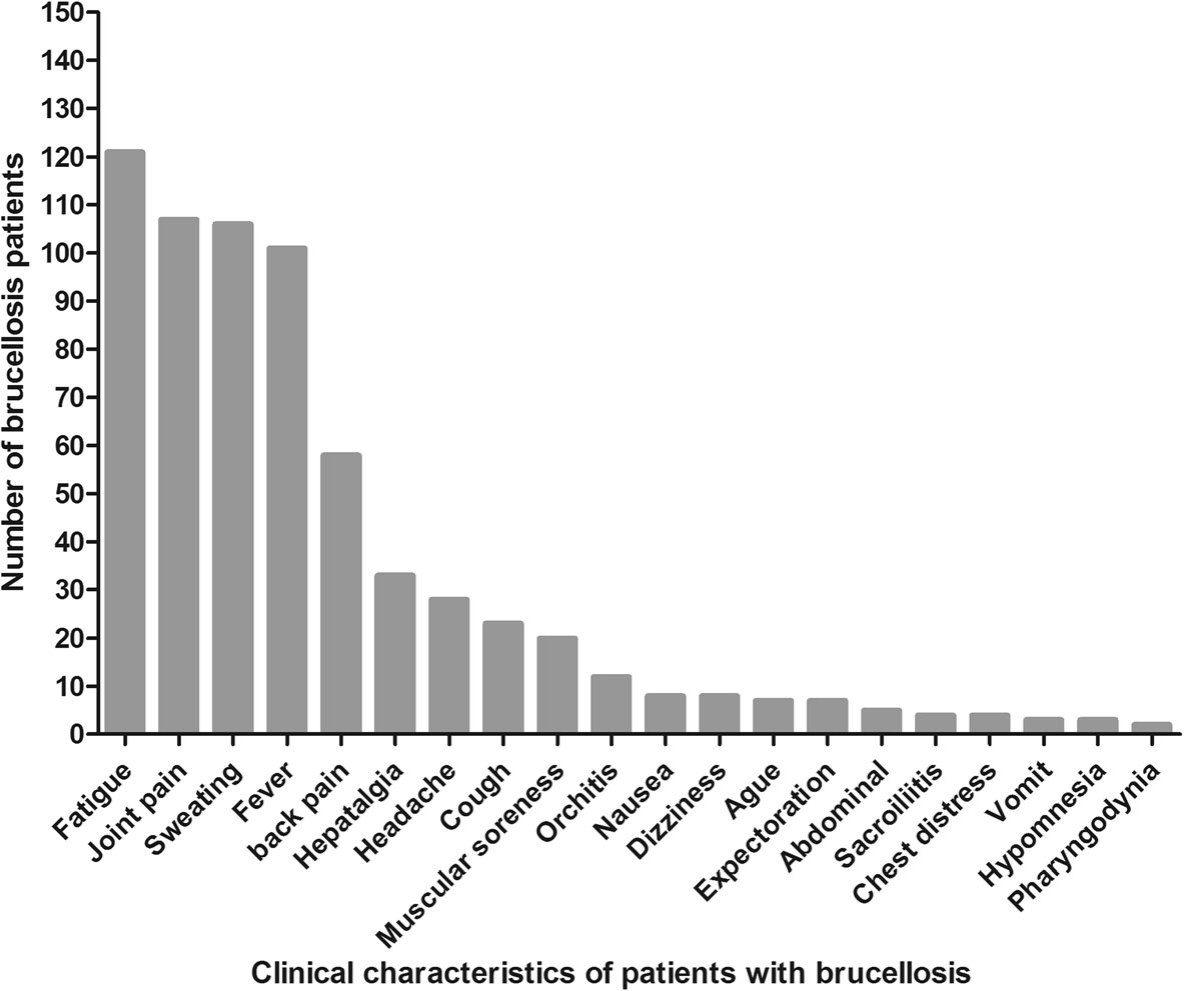
Clinical features of patients with brucellosis

Simultaneously, more than 70% patients presented four typical clinical symptoms: fatigue, joint pain, sweating and fever (Fig. 2). In addition, backache, hepatalgia, headache, muscular soreness and orchitis appeared relatively common. Only 10 patients experienced nausea, vomiting, anorexia and other symptoms (Fig. 2). Brucellosis was diagnosed by a combination of clinical examination and laboratory testing with RBPT and SAT at admission to hospital. Some of these patients had received antimicrobial treatment, while others remained untreated (Additional file 1: Table S1).

### Detection of *Brucella* antigens by IFS in PBMCs of treated brucellosis patients

**Fig. 3:**
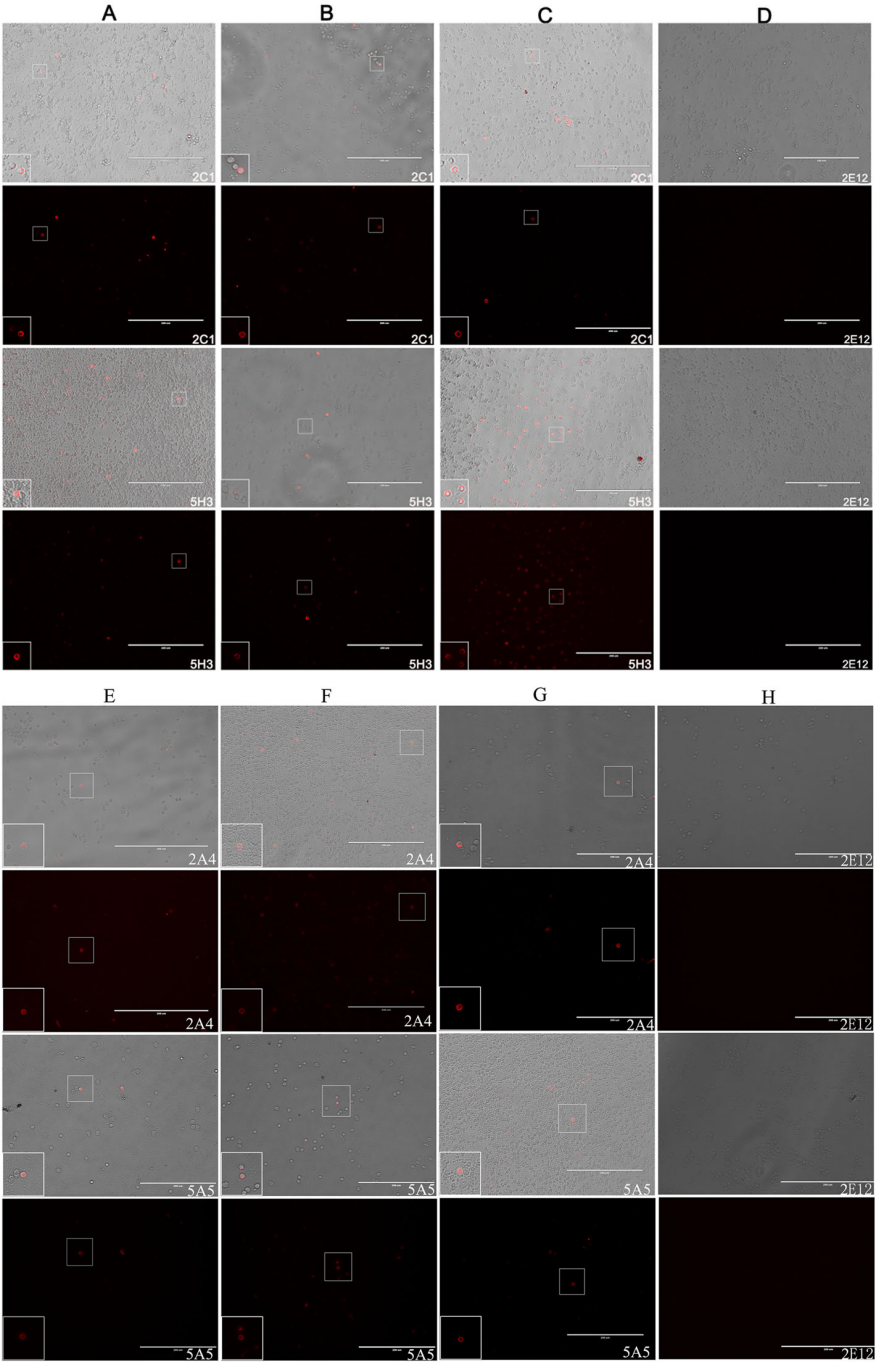
Detection of *Brucella* antigen in PBMCs by IFS with mAbs. (**a**, **b**, **c**, **e**, **f**, **g**) Brucellosis patients’ PBMCs were stained by IFS with mAbs 2C1 and 5H3 specific to Omp31 antigens, 2A4 and 5A5 specific to Bp26 antigens of *Brucella* species, individually; (**d**, **h**) The NS3 antibody 2E12 for HCV was reactive as negative control. IFS stained cells were examined by a Nikon Labophot photomicroscope under white or fluorescent light. Scale bars = 200 μm

*Brucellae* are known for persistently residing within phagocytes in chronic brucellosis patients and orchestrating diverse immune evasion strategies from this niche [4, 13]. Once infected with *Brucellae*, most patients show symptoms during the acute phase and could be either cured or progressing to chronicity with long symptomatic periods. In this study, the PBMCs from 154 patients and 36 blood donors were tested for intracellular *Brucellae* by IFS with mAbs 2C1, 5H3, 2A4 and 5A5 specific to either *Brucella* BP26 or OMP31 protein, respectively. Pictures of visible intracellular *Brucellae* were taken with a fluorescence microscope with appropriate filters (Fig. 3). Overall, 68.8% (106/154) patients carried intra-PBMC bacteria (Table 1), while all 36 blood donors were negative by IFS.

**Table 1:** Reactivity of *Brucella* antibody or antigen in brucellosis patients

	Reactive	Number (%)	IFS	
			+ (%)	- (%)
Patient number		154 (100)	106 (68.8)	48 (31.2)
RBPT	+	92 (59.7)	66 (42.8)	26 (16.9)
	–	62 (40.3)	40 (26)	22 (14.3)
SAT	+	126 (81.8)	87 (56.5)	39 (25.3)
	–	28 (18.1)	19 (12.3)	9 (5.8)
ELISA	+	148 (96.1)	103 (66.9)	45 (29.2)
	–	6 (3.8)	3 (1.9)	3 (1.9)
Total^a^	+	151 (98)	104 (67.5)	47 (30.5)
	–	3 (1.9)	2 (1.3)	1 (0.6)
Bacterial Culture	+	5 (3.2)	4 (2.6)	1 (0.6)
	–	149 (96.8)	102 (66.3)	47 (30.5)

^a^Compiled results of three serological testing methods and IFS

### Comparison of detection rates between antibody and antigen tests in hospitalized patients with brucellosis

Blood samples from 154 brucellosis patients were re-tested during hospitalization. Data showed 59.7% (92/154) of blood samples antibody reactive with RBPT, 81.8% (126/154) with SAT, and 96.1% (148/154) with ELISA, respectively. Only 3.2% (5/154) of bacterium blood cultures were positive compared with 68.8% (106/154) PBMCs *Brucella* antigen positive by IFS. Moreover, 22 (14.3%) patients were negative with both IFS and RBPT, and 9 (5.8%) with both IFS and SAT. Antigen detection was negative with both IFS and culture in 47 patients (30.5%). Altogether only one patient (0.6%) was negative with all serologic and antigen tests (Table 1).

Compared with antibody detection, 72.2% (65/90) samples were positive by IFS and reacted with all three serologic methods (Table 2). Several cases were negative for one or two serologic tests but positive for IFS. For example, 68% (17/25) samples positive with IFS were negative for both RBPT and SAT. Among ELISA or RBPT negative samples, 50% (1/2) or 63.6% (21/33) reacted with IFS, respectively (Table 2). Only two patients (1.3%) were antibody test negative but positive for bacterial antigens by IFS (Table 1), suggesting that patients with persistent *Brucella* in PBMCs may lose detectable antibody reactivity. Patients with negative IFS (47/154 or 30.5%, Table 1) might have effectively eliminated the bacteria as a result of treatment.

**Table 2:** Comparison of detection rate between *Brucella* antigen and antibody

RBPT	SAT	ELISA	Intra-cellular *Brucella* antigen by IFS		Patient number
			Positive (%)	Negative (%)	Total
Pos	Pos	Pos	65 (72.2)	25 (27.8)	90
Pos	Pos	Neg	1 (50)	1 (50)	2
Pos	Neg	Pos	0	0	0
Neg	Pos	Pos	21 (63.6)	12 (36.4)	33
Pos	Neg	Neg	0	0	0
Neg	Pos	Neg	0	1 (100)	1
Neg	Neg	Pos	17 (68)	8 (32)	25
Neg	Neg	Neg	2 (66.7)	1 (33.3)	3
Overall			106 (68.8)	48 (31.2)	154

### Association of IFS detection with potential factors of brucellosis

We examined the correlation between detection rate of *Brucellae* and multiple potentially associated factors. Only gender was significantly correlated with IFS detection (*P* = 0.01), others factors such as the age, therapeutic management and stage of disease were unrelated to detectable intracellular *Brucellae* (*P* > 0.05) (Table 3). There was no significant difference in the rate of PBMC bacterium carriers between patients with acute or chronic brucellosis (70.6 and 67.4%, respectively, *P* > 0.05). The IFS rate of intracellular *Brucellae* detection in PBMCs was not significantly reduced by any particular treatment regimen. Treated patients were 68.7% IFS positive, nearly equal to the pre-treatment 68.8%. Positive PBMC rates remained similar when patients received antimicrobial drugs alone (79.5%) or drugs combined with immune enhancing agents (63.8%) (Table 3).

**Table 3:** Factors potentially impacting IFS detection of human brucellosis

		IFS (Nb/%)		Total	*Ρ* value
		+	–		
Gender*	Male	72 (63.2%)	42 (36.8%)	114	0.01
	Female	34 (85%)	6 (15%)	40	
Status	Acute	48 (70.6%)	20 (29.4%)	68	0.676
	Chronic	58 (67.4%)	28 (32.6%)	86	
Treatment	Treated	90 (68.7%)	41 (31.3%)	131	0.934
	Untreated	16 (69.9%)	7 (30.4%)	23	
Therapy regimen^#Δ^	Routine	31 (79.5%)	8 (20.5%)	39	0.088
	Addition	44 (63.8%)	25 (36.2%)	69	
Mean age	43.4 ± 13.5	43.9 (68.8%)	42.2 (31.2%)	154	0.472

*P value of less than 0.05 was considered significant

#Routine therapy regimen indicated the standard antibiotic treatment; Additional therapy regimen indicated the standard antibiotic treatment additionally receiving immune enhancing drugs

ΔTherapy regimen situation of 23 treated brucellosis patients was missing

### Significance of intracellular *Brucellae* detection in monitoring therapeutic efficacy in acute or chronic brucellosis patients

Among 154 brucellosis patients, 68 were classified as acute phase of infection. The antibody or antigen detection showed that 98.5% (67/68) and 70.6% (48/68) were positive with SAT or IFS, respectively (Table 3). Treated acute brucelloses presented 84.4% positive IFS after the first two treatment courses and 53.8% positive test after the third course, suggesting 30% a significant difference of therapeutic efficacy (*P* = 0.011). This difference was not observed with antibody detection by SAT and ELISA (Table 4), indicating that reactive antibody against *Brucellae* might persist for long period after bacteria had been eliminated. The completion of a third therapeutic course appeared as a key element to ensure a favorable outcome. Generally most acute brucellosis patients were discharged from hospital after the third course of therapy, although more than half of them (53.8%) still carried intra-PBMC *Brucellae* (Table 4), suggesting that further treatment might remain indicated. In contrast, patients with chronic brucellosis showed no difference (*P* > 0.05) between SAT or ELISA for antibody and IFS for bacteria, irrespective of treatment regimen (Table 4).

**Table 4:** Comparison of antibody and antigen detection rates in acute or chronic brucellosis

Phase	Therapy course		Reactivity (Nb/%)		Total	*P* value Intragroup
			+	–		
Acute	SAT	0	10 (100%)	0	10	0.467
		1–2	31 (96.9%)	1 (3.1%)	32	
		≥3	26 (100%)	0	26	
		Overall	67 (98.5%)	1 (1.5%)	68	
	ELISA	0	9 (90%)	1 (10%)	10	0.209
		1–2	30 (93.8%)	2 (6.2%)	32	
		≥3	26 (100%)	0	26	
		Overall	65 (95.6%)	3 (4.4%)	68	
	IFS	0	7 (70%)	3 (30%)	10	0.038*
		1–2	27 (84.4%)	5 (15.6%)	32	
		≥3	14 (53.8%)	12 (46.2%)	26	
		Overall	48 (70.6%)	20 (29.4%)	68	
Chronic	SAT	0	12 (92.3%)	1 (7.7%)	13	0.061
		1–2	11 (73.3%)	4 (26.7%)	15	
		≥3	36 (62.1%)	22 (37.9%)	58	
		Overall	59 (68.6%)	27 (31.4%)	86	
	ELISA	0	13 (100%)	0	13	0.299
		1–2	15 (100%)	0	15	
		≥3	55 (94.8%)	3 (5.2%)	58	
		Overall	83 (96.5%)	3 (3.5%)	86	
	IFS	0	9 (69.2%)	4 (30.8%)	13	0.988
		1–2	10 (66.7%)	5 (33.3%)	15	
		≥3	39 (67.2%)	19 (32.8%)	58	
		Overall	58 (67.4%)	28 (32.6%)	86	

*A significant difference was found in IFS detection rate among therapy courses 0, 1–2 and ≥ 3 (*P* = 0.038), or between therapy courses 0–1-2 and ≥ 3 (*P* = 0.017) or 1–2 and ≥ 3 (*P* = 0.011) from acute phase of brucellosis patients

## Discussion

Brucellosis is a highly contagious zoonosis. Epidemiological study showed that the main risk factors included contacts with infected animals, ingestion of unpasteurized milk and dairy products. Recently, a review indicated that 24.6% of 590 brucellosis patients, in Xin Jiang province of China, were infected through consumption of raw and uncooked animal products. Among 154 patients ranging between 3 to 73 years of both genders in this study (Fig. 1 and Additional File 1: Table S1), only 3.9% were infected through eating raw or uncooked animal products. This percentage was lower than reported in other countries, such as 62.6–94.6% in Turkey and 79.1% in Iran [12], presumably indicating different traditional eating habits. Additionally, most patients in the present study (approximately 90.3%) were infected through close contacts with animals as previously described [12], and their occupations did carry risk of infection [14, 15]. Gender (male 74.0% and female 26.0%) was correlated with the rate of positive IFS, but people working as farmer, breeder or veterinarian, were mostly male. There was a common cause of brucellosis transmission in Heilongjiang and Xinjiang provinces that drew attention to the prevention of human brucellosis. Animals are frequently transported across China [16] and more and more Chinese people travel across the country. Moreover, many people eat undercooked local dishes, such as roast lamb and other related food in Xinjiang, Inner Mongolia and other northern areas of China. These Northern provinces are the main epidemic areas of brucellosis.

Most clinical symptoms of human brucellosis are unspecific. In this study 70% of patients experienced fatigue, arthralgia, hyperhydrosis and fever (Fig. 2). Less frequent were backache, hepatalgia, headache, muscular soreness and orchitis that appeared relatively unspecific. Few patients had other symptoms including nausea, vomiting and decreased appetite. Considering the lack of typical clinical manifestations and extremely slow growth of *Brucellae*, it was difficult to distinguish brucellosis from other sickness with similar clinical symptoms contributing to misdiagnosis and delay in diagnosis and therapy [14, 15]. In addition, relapse of brucellosis was sometimes difficult to diagnose, considering the frequency of false negative blood cultures (definite diagnosis of brucellosis), and how ubiquitous symptoms were [17]. Among 154 patients in our study, only 3.2% blood samples were positive by culture (Table 1), a percentage much lower than in other reports [2] contrasting with 68.8% detection of intra-cellular *Brucella* antigens. Due to high infectivity of *Brucellae*, blood culture carries a risk to laboratory staff. The diagnosis of brucellosis traditionally relies on serologic testing, such as RBPT, SAT and ELISA and other methods. Each of these serologic assays having relatively poor performance a direct assay for *Brucella* antigen is needed for accurate diagnosis. In this study 154 patients were retrospectively found positive 59.7% with RBPT, 81.8% with SAT and 96.1% with ELISA, respectively (Table 1), consistent with previous reports that ELISA had superior performance. It was therefore the recommended method for the diagnosis of infection with *Brucella abortus, melitensis* and *suis* by OIE [18] that may replace other antibody tests in the future [19]. Additionally, we found 47 patients (30.5%) negative with IFS but positive by antibody detection whose antibody persisted for prolonged periods of time (Table 1). However, chronic patients were frequently negative for SAT (27/86, 31.4%) but few in acute patients (1/68, 1.5%) suggesting that antibody reactivity might become undetectable as patients progressed to chronic infection (Table 4). For example, we observed several cases negative for one or more serologic tests but positive for IFS (Table 2). In addition, 68% (17/25) of both SAT and RBPT negative patients remained positive by IFS (Table 2). Treated patients with acute or chronic brucellosis were not significantly different in terms of antibody detection with SAT and ELISA, irrespective of treatment courses (Table 4). Therefore, serologic tests appeared rather unsuitable for patient follow-up, and were not always informative for latent carriers of *Brucella* infection [20–22]. Antibody reactivity or titer were not reliable laboratory tests to estimate the efficacy of treatment or passage to chronicity. During the course of therapy, physicians might estimate treatment efficacy according to persistence of symptoms and presence of intra-cellular *Brucellae* detected by IFS.

For this purpose, IFS was developed to detect *Brucellae* [7] and was extensively used in this study. IFS identified 68.8% (106/154) of patient PBMCs carrying the bacterial antigen massively increasing direct detection of *Brucellae* in patients’ PBMCs without the specificity issues inherent to serologic assays. The detection rate of *Brucella* antigens was not related to patient’s age or treatment regimen (*P* > 0.05). The significant infection risk related to gender (*P* = 0.01) was unsurprising since the male population carries most occupational or professional activities involving close contact with infected animals.

The frequency of intracellular *Brucellae* in PBMCs of infected patients was similar whether they were in acute or chronic state or receiving different treatment regimens (*P* > 0.05). It is well known that brucellosis seldom recovers completely, which coincides with high detection rate by IFS in 69.9% of untreated brucellosis and 68.7% of treated patients (Table 3). Once the infection becomes chronic, patients are likely to keep carrying *Brucellae* for their whole life, as happened to our 58 chronic patients ranging in age from 4 to 73 years who received more than three courses of therapy but a majority (67.2%) remained IFS positive (Table 4). At the chronic stage test positivity was even higher, 62.1% by SAT or 94.8% by ELISA (Table 4). Unfortunately the study was strictly cross-sectional since sequential samples could only be obtained for two patients.

Despite the lack of correlation with positive culture, our IFS intra-cellular *Brucellae* antigen strongly suggests the prolonged persistence of the bacterium in chronic cases, irrespective of therapy (Fig. 3). It was previously reported that patients had a tendency toward chronicity and persistence, becoming a granulomatous disease capable of affecting any organ [2, 23]. However, it would be critical to determine whether or not the 31.2% patients with negative IFS were effectively free of the infection either spontaneously or through combination therapy. Answer to this question would require sequential IFS testing of patients with chronic infection with and without symptoms, with and without therapy. The 67.4% (58/86) of patients with chronic infection positive by IFS illustrate the difficulty of eliminating intracellular *Brucellae* and the poor efficacy of current treatments for chronic brucellosis. This situation is supported by previous description of *Brucella* chronic infection [10, 24]. There is a need for novel drugs to improve the efficacy of treatments that might be monitored with the IFS assay.

The poor therapeutic outcome of therapy in chronic brucellosis might be mitigated by the data obtained in acute infection treatment. While antibody remained positive, all except one patient having received two courses of treatment had significantly higher prevalence of positive IFS assay than those having received a third course of therapy in hospital (Table 4). Such difference observed in relatively small groups of patients studied transversally would need confirmation in a proper longitudinal study. The IFS assay might, at this point, represent the most suitable means for evaluating brucellosis treatment. It might be equally important to combine the IFS with other antibody tests. In this study, only 14.3% (22/154) were negative with both IFS and RBPT, and 5.8% (9/154) with both IFS and SAT. Compared to 3.2% positive rate of blood culture, 102/149 patients (66.3%) carried PBMCs with detectable bacteria with the IFS technique (Table 1), indicating a massive improvement in the detection of intracellular *Brucellae*. Considering the biosafety requirements, the time-consuming aspect of culture of *Brucella* strains, IFS detection of intracellular *Brucellae* might be a better diagnostic or monitoring test of brucellosis treatment in clinical practice.

Several patients in the study received immunopotentiators in addition to the standard antibiotic treatment. There was no clear benefit of this approach evidenced by a significant decrease of IFS positivity (*P* > 0.05) (Table 3). Some data in our earlier study may provide some clue. Specifically, the ratio of regulatory T-lymphocytes cell (CD3^+^CD4^+^Foxp3^+^ Treg) in PBMCs of brucellosis patients determined by flow cytometry before and after treatment showed that after the first and second courses of treatment, the frequency of Foxp3^+^ Tregs did not significantly change, as the frequency was still higher than that in healthy populations, and there was no significant difference between untreated and treated groups [25]. After the third course of treatment (about 80 days), the frequency of Foxp3^+^ Tregs in the PBMCs of acute brucellosis patients decreased to normal level of healthy individuals, while the frequency of Foxp3^+^ Tregs in immune enhancing treatment patients remained higher. The data presented here suggests that the antibacterial treatment of brucellosis should benefit of including at least three courses of treatment and that the IFS assay could be used to monitor therapeutic efficacy.

## Conclusions

There is a lack of effective methods to detect *Brucella* antigen and monitor treatment. In our study, 68.8% PBMCs of brucellosis patients contained *Brucellae* detectable by IFS. A significant decrease of intracellular *Brucellae* detected by IFS was only observed in acute brucellosis patients after the third course of treatment, suggesting that 30% of therapeutic efficacy and current therapeutic regimens were not effective for chronic brucellosis. There was no significant difference in antibody detection with SAT and ELISA, irrespective of treatment courses. IFS appears a sensitive assay for detection of *Brucella* antigen in PBMCs and could be used for diagnosis and therapeutic monitoring of brucellosis in clinical practice.

## Supplementary information

**Additional file 1: Table S1.** Characterization of 154 brucellosis patients.

## Data Availability

All data and materials associated with this study are available from the main text or the additional file. The monoclonal antibodies to *Brucellae* are available in the Department of Transfusion Medicine, Southern Medical University, Guangzhou, China.

## Abbreviations

ELISA: enzyme-linked immunosorbent assay
IFS: immunofluorescence staining
PBMC: peripheral blood mononuclear cell
RBPT: Rose Bengal plate test
SAT: standard tube agglutination test
