# Supplementary material for: Detection of Brucellae in peripheral blood mononuclear cells for monitoring therapeutic efficacy of brucellosis infection
Source: Antimicrob Resist Infect Control. 2019 Oct 15;8:154. doi: 10.1186/s13756-019-0607-2 (PMC6794757; doi:10.1186/s13756-019-0607-2)
Supplement: Supplementary file 1 — Additional file 1: Table S1. Characterization of 154 brucellosis patients. [file 13756_2019_607_MOESM1_ESM.doc]

**Additional file 1: Table S1 Characterization of 154 brucellosis patients**

| Patients | Gender | Age | Occupation | Epidemiology history | Stage ^Δ^ | Therapy course * | Treatment ^#^ | IFS | RBPT | ELISA | SAT | Culture | |
| --- | --- | --- | --- | --- | --- | --- | --- | --- | --- | --- | --- | --- | --- |
|  | Female | 3 | Student | Exposure and farming of cattle or sheep | Acute | ≥3 | Antimicrobial | + | + | + | 1:200 | | - |
|  | Female | 4 | Student | Exposure and farming of cattle or sheep | Acute | ≥3 | Immune enhancing agents | + | + | + | 1:800 | | - |
|  | Male | 4 | Student | Consumption history of raw meat or dairy | Chronic | ≥3 | Immune enhancing agents | + | + | + | 1:400 | | - |
|  | Male | 5 | Student | Exposure and farming of cattle or sheep | Acute | ≥3 | Immune enhancing agents | + | + | + | 1:400 | | - |
|  | Male | 6 | Farmer | Exposure and farming of cattle or sheep | Acute | ≥3 | Immune enhancing agents | + | + | + | 1:400 | | - |
|  | Male | 9 | Student | Exposure and farming of cattle or sheep | Chronic | 0 | Untreated | - | - | + | 1:800 | | - |
|  | Male | 16 | Student | Exposure and farming of cattle or sheep | Chronic | ≥3 | Antimicrobial | + | + | + | 1:200 | | - |
|  | Male | 21 | Farmer | Exposure and farming of cattle or sheep | Acute | ≥3 | Antimicrobial | + | + | + | 1:800 | | - |
|  | Male | 21 | Veterinarian | Exposure and farming of cattle or sheep | Acute | ≥3 | Immune enhancing agents | + | + | + | 1:200 | | - |
|  | Male | 21 | Farmer | Exposure and farming of cattle or sheep | Acute | ≥3 | Immune enhancing agents | - | - | + | 1:50 | | - |
|  | Male | 22 | Stockman | Exposure and farming of cattle or sheep | Acute | ≥3 | Immune enhancing agents | - | + | + | 1:100 | | - |
|  | Female | 23 | Farmer | Exposure and farming of cattle or sheep | Acute | 1-2 | Antimicrobial | + | + | + | 1:400 | | - |
|  | Male | 23 | Veterinarian | Exposure and farming of cattle or sheep | Chronic | ≥3 | Antimicrobial | + | + | + | 1:100 | | - |
|  | Male | 24 | Farmer | Exposure and farming of cattle or sheep | Acute | ≥3 | Antimicrobial | - | + | + | 1:100 | | - |
|  | Male | 24 | Farmer | Exposure and farming of cattle or sheep | Acute | 1-2 | Antimicrobial | + | + | + | 1:100 | | - |
|  | Male | 25 | Stockman | Exposure and farming of cattle or sheep | Acute | 1-2 | Unknown | + | + | + | 1:100 | | - |
|  | Male | 25 | Farmer | Exposure and farming of cattle or sheep | Chronic | ≥3 | Unknown | + | - | + | — | | - |
|  | Male | 26 | Farmer | Exposure and farming of cattle or sheep | Acute | 1-2 | Immune enhancing agents | + | + | + | 1:400 | | - |
|  | Male | 26 | Veterinarian | Exposure and farming of cattle or sheep | Acute | 1-2 | Antimicrobial | - | + | + | 1:100 | | - |
|  | Male | 26 | Veterinarian | Exposure and farming of cattle or sheep | Acute | 1-2 | Antimicrobial | - | + | + | 1:100 | | - |
|  | Female | 26 | Stockman | Exposure and farming of cattle or sheep | Acute | 1-2 | Unknown | + | - | - | — | | - |
|  | Male | 27 | Veterinarian | Exposure and farming of cattle or sheep | Chronic | 0 | Untreated | + | + | + | 1:100 | | - |
|  | Male | 28 | Veterinarian | Exposure and farming of cattle or sheep | Chronic | ≥3 | Immune enhancing agents | - | - | + | 1:50 | | - |
|  | Male | 29 | Veterinarian | Exposure and farming of cattle or sheep | Chronic | ≥3 | Immune enhancing agents | - | - | + | 1:50 | | - |
|  | Male | 31 | Veterinarian | Exposure and farming of cattle or sheep | Chronic | ≥3 | Immune enhancing agents | + | + | + | 1:50 | | - |
|  | Male | 31 | Veterinarian | Exposure and farming of cattle or sheep | Chronic | ≥3 | Immune enhancing agents | - | - | + | 1:50 | | - |
|  | Male | 32 | Veterinarian | Exposure and farming of cattle or sheep | Chronic | 0 | Untreated | + | + | + | 1:100 | | - |
|  | Male | 33 | Veterinarian | Exposure and farming of cattle or sheep | Acute | ≥3 | Immune enhancing agents | + | + | + | 1:100 | | - |
|  | Male | 34 | Veterinarian | Exposure and farming of cattle or sheep | Chronic | 0 | Untreated | - | + | + | 1:100 | | - |
|  | Male | 34 | Farmer | Exposure and farming of cattle or sheep | Acute | 0 | Untreated | - | + | + | 1:400 | | - |
|  | Female | 35 | Farmer | Exposure and farming of cattle or sheep | Acute | 1-2 | Antimicrobial | + | + | + | 1:400 | | - |
|  | Male | 35 | Farmer | Exposure and farming of cattle or sheep | Acute | 1-2 | Antimicrobial | + | + | + | 1:400 | | + |
|  | Male | 36 | Veterinarian | Exposure and farming of cattle or sheep | Chronic | ≥3 | Immune enhancing agents | + | + | + | 1:100 | | - |
|  | Male | 36 | Farmer | Unknown | Acute | ≥3 | Unknown | + | - | + | 1:50 | | + |
|  | Female | 36 | Farmer | Unknown | Chronic | ≥3 | Unknown | - | - | + | 1:50 | | - |
|  | Male | 37 | Stockman | Exposure and farming of cattle or sheep | Acute | 1-2 | Antimicrobial | + | + | + | 1:200 | | - |
|  | Female | 37 | Stockman | Exposure and farming of cattle or sheep | Chronic | ≥3 | Immune enhancing agents | + | - | + | 1:50 | | - |
|  | Male | 38 | Farmer | Exposure and farming of cattle or sheep | Acute | ≥3 | Immune enhancing agents | - | + | + | 1:100 | | - |
|  | Male | 38 | Veterinarian | Exposure and farming of cattle or sheep | Chronic | ≥3 | Antimicrobial | + | + | + | 1:100 | | - |
|  | Male | 38 | Stockman | Exposure and farming of cattle or sheep | Acute | 1-2 | Antimicrobial | + | - | + | 1:50 | | - |
|  | Male | 38 | Veterinarian | Exposure and farming of cattle or sheep | Chronic | 0 | Untreated | + | - | + | 1:100 | | - |
|  | Male | 39 | Farmer | Exposure and farming of cattle or sheep | Acute | ≥3 | Immune enhancing agents | - | + | + | 1:200 | | - |
|  | Male | 39 | Farmer | Exposure and farming of cattle or sheep | Chronic | ≥3 | Immune enhancing agents | - | + | + | 1:100 | | - |
|  | Male | 39 | Farmer | Exposure and farming of cattle or sheep | Chronic | ≥3 | Unknown | - | + | + | 1:400 | | - |
|  | Male | 39 | Farmer | Exposure and farming of cattle or sheep | Chronic | ≥3 | Immune enhancing agents | + | + | + | 1:100 | | - |
|  | Male | 40 | Veterinarian | Exposure and farming of cattle or sheep | Acute | ≥3 | Immune enhancing agents | - | + | + | 1:400 | | - |
|  | Female | 40 | Stockman | Exposure and farming of cattle or sheep | Chronic | ≥3 | Unknown | + | - | - | — | | - |
|  | Male | 40 | Breeder | Exposure and farming of cattle or sheep | Chronic | 1-2 | Antimicrobial | + | - | + | — | | - |
|  | Male | 40 | Veterinarian | Exposure and farming of cattle or sheep | Chronic | ≥3 | Immune enhancing agents | - | - | - | — | | - |
|  | Male | 41 | Farmer | Exposure and farming of cattle or sheep | Acute | 1-2 | Antimicrobial | + | + | + | 1:800 | | - |
|  | Male | 41 | Veterinarian | Exposure and farming of cattle or sheep | Chronic | ≥3 | Immune enhancing agents | - | + | + | 1:100+ | | - |
|  | Male | 41 | Stockman | Exposure and farming of cattle or sheep | Chronic | 1-2 | Antimicrobial | - | + | + | 1:100 | | - |
|  | Female | 41 | Farmer | Exposure and farming of cattle or sheep | Chronic | ≥3 | Immune enhancing agents | + | - | + | — | | - |
|  | Male | 42 | Veterinarian | Exposure and farming of cattle or sheep | Acute | 1-2 | Immune enhancing agents | + | + | + | 1:200 | | - |
|  | Male | 42 | Stockman | Exposure and farming of cattle or sheep | Acute | ≥3 | Immune enhancing agents | - | + | + | 1:100 | | - |
|  | Male | 42 | Farmer | Exposure and farming of cattle or sheep | Acute | ≥3 | Immune enhancing agents | - | + | + | 1:200 | | - |
|  | Male | 42 | Veterinarian | Exposure and farming of cattle or sheep | Chronic | 0 | Untreated | - | + | + | 1:50 | | - |
|  | Male | 42 | Veterinarian | Exposure and farming of cattle or sheep | Chronic | ≥3 | Unknown | + | - | + | — | | - |
|  | Male | 42 | Veterinarian | Exposure and farming of cattle or sheep | Chronic | ≥3 | Immune enhancing agents | - | - | - | 1:50 | | - |
|  | Female | 42 | Farmer | Unknown | Chronic | ≥3 | Unknown | + | - | + | — | | - |
|  | Female | 43 | Farmer | Exposure and farming of cattle or sheep | Acute | 0 | Untreated | + | + | + | 1:100 | | - |
|  | Male | 43 | Farmer | Exposure and farming of cattle or sheep | Chronic | 1-2 | Antimicrobial | - | + | + | 1:200 | | - |
|  | Male | 43 | Veterinarian | Exposure and farming of cattle or sheep | Chronic | ≥3 | Immune enhancing agents | - | - | + | — | | - |
|  | Female | 43 | Farmer | Unknown | Chronic | 0 | Untreated | + | - | + | 1:50 | | - |
|  | Male | 43 | Veterinarian | Exposure and farming of cattle or sheep | Chronic | ≥3 | Immune enhancing agents | + | - | + | — | | - |
|  | Male | 43 | Veterinarian | Exposure and farming of cattle or sheep | Chronic | ≥3 | Unknown | - | - | + | 1:50 | | - |
|  | Male | 43 | Farmer | Exposure and farming of cattle or sheep | Acute | 1-2 | Immune enhancing agents | + | - | + | 1:50 | | - |
|  | Male | 43 | Veterinarian | Exposure and farming of cattle or sheep | Chronic | 1-2 | Unknown | - | - | + | 1:50 | | - |
|  | Male | 44 | Farmer | Exposure and farming of cattle or sheep | Acute | 1-2 | Antimicrobial | + | + | + | 1:1600 | | + |
|  | Male | 44 | Farmer | Consumption history of raw meat or dairy | Acute | 0 | Untreated | - | + | + | 1:200 | | - |
|  | Male | 44 | Stockman | Exposure and farming of cattle or sheep | Chronic | ≥3 | Immune enhancing agents | + | + | + | 1:100 | | - |
|  | Female | 44 | Farmer | Exposure and farming of cattle or sheep | Chronic | ≥3 | Unknown | + | - | + | — | | - |
|  | Male | 44 | Farmer | Unknown | Chronic | 0 | Untreated | + | - | + | 1:50 | | - |
|  | Male | 45 | Farmer | Exposure and farming of cattle or sheep | Chronic | ≥3 | Antimicrobial | + | + | + | 1:100 | | - |
|  | Male | 45 | Small business owner | Exposure and farming of cattle or sheep | Acute | ≥3 | Immune enhancing agents | + | + | + | 1:800 | | - |
|  | Male | 45 | Veterinarian | Exposure and farming of cattle or sheep | Chronic | ≥3 | Immune enhancing agents | + | - | + | — | | - |
|  | Female | 45 | Farmer | Exposure and farming of cattle or sheep | Chronic | ≥3 | Immune enhancing agents | + | - | + | 1:50 | | - |
|  | Male | 45 | Veterinarian | Exposure and farming of cattle or sheep | Chronic | ≥3 | Immune enhancing agents | - | - | + | 1:50 | | - |
|  | Male | 46 | Farmer | Exposure and farming of cattle or sheep | Acute | 1-2 | Antimicrobial | + | + | + | 1:100 | | - |
|  | Male | 46 | Veterinarian | Exposure and farming of cattle or sheep | Acute | ≥3 | Immune enhancing agents | + | + | + | 1:200 | | - |
|  | Male | 46 | Stockman | Exposure and farming of cattle or sheep | Acute | 1-2 | Immune enhancing agents | + | + | + | 1:100 | | - |
|  | Male | 46 | Farmer | Unknown | Acute | ≥3 | Unknown | - | + | + | 1:200 | | - |
|  | Male | 46 | Farmer | Unknown | Acute | ≥3 | Unknown | - | + | + | 1:200 | | - |
|  | Male | 46 | Veterinarian | Exposure and farming of cattle or sheep | Chronic | 1-2 | Immune enhancing agents | + | + | + | 1:200 | | - |
|  | Female | 46 | Farmer | Exposure and farming of cattle or sheep | Chronic | ≥3 | Immune enhancing agents | + | - | + | — | | - |
|  | Male | 46 | Farmer | Exposure and farming of cattle or sheep | Chronic | ≥3 | Immune enhancing agents | + | - | + | — | | - |
|  | Male | 46 | Stockman | Exposure and farming of cattle or sheep | Chronic | ≥3 | Unknown | + | - | + | 1:50 | | - |
|  | Male | 46 | Veterinarian | Exposure and farming of cattle or sheep | Chronic | ≥3 | Immune enhancing agents | + | - | + | 1:100 | | - |
|  | Male | 47 | Stockman | Consumption history of raw meat or dairy | Acute | 1-2 | Antimicrobial | - | + | + | 1:1600 | | - |
|  | Male | 47 | Veterinarian | Exposure and farming of cattle or sheep | Chronic | 1-2 | Unknown | + | - | + | — | | - |
|  | Male | 47 | Veterinarian | Exposure and farming of cattle or sheep | Chronic | ≥3 | Unknown | + | - | + | 1:50 | | - |
|  | Male | 48 | Farmer | Exposure and farming of cattle or sheep | Acute | 0 | Untreated | - | + | - | 1:400 | | + |
|  | Male | 48 | Farmer | Exposure and farming of cattle or sheep | Acute | 1-2 | Antimicrobial | + | + | + | 1:400 | | - |
|  | Male | 48 | Veterinarian | Exposure and farming of cattle or sheep | Chronic | 1-2 | Antimicrobial | + | - | + | — | | - |
|  | Female | 48 | Farmer | Exposure and farming of cattle or sheep | Chronic | ≥3 | Immune enhancing agents | + | - | + | 1:400 | | - |
|  | Female | 48 | Farmer | Exposure and farming of cattle or sheep | Chronic | ≥3 | Immune enhancing agents | - | - | + | — | | - |
|  | Female | 48 | Farmer | Exposure and farming of cattle or sheep | Chronic | 0 | Untreated | + | - | + | 1:50 | | - |
|  | Male | 48 | Farmer | Exposure and farming of cattle or sheep | Chronic | 1-2 | Immune enhancing agents | - | - | + | 1:50 | | - |
|  | Male | 49 | Farmer | Exposure and farming of cattle or sheep | Chronic | 1-2 | Antimicrobial | + | + | + | 1:100 | | - |
|  | Male | 49 | Veterinarian | Exposure and farming of cattle or sheep | Chronic | 1-2 | Antimicrobial | + | - | + | 1:50 | | - |
|  | Male | 49 | Stockman | Exposure and farming of cattle or sheep | Chronic | ≥3 | Unknown | - | - | + | 1:50 | | - |
|  | Male | 49 | Veterinarian | Exposure and farming of cattle or sheep | Chronic | ≥3 | Immune enhancing agents | + | - | + | — | | - |
|  | Female | 50 | Farmer | Exposure and farming of cattle or sheep | Acute | 1-2 | Antimicrobial | - | + | + | 1:100 | | - |
|  | Male | 50 | Stockman | Exposure and farming of cattle or sheep | Acute | 1-2 | Immune enhancing agents | + | + | + | 1:200 | | - |
|  | Female | 50 | Farmer | Exposure and farming of cattle or sheep | Chronic | 0 | Untreated | + | + | + | 1:200 | | - |
|  | Female | 50 | Stockman | Exposure and farming of cattle or sheep | Chronic | ≥3 | Immune enhancing agents | + | + | + | 1:400 | | - |
|  | Male | 50 | Stockman | Exposure and farming of cattle or sheep | Chronic | ≥3 | Immune enhancing agents | + | + | + | 1:100 | | - |
|  | Male | 50 | Veterinarian | Exposure and farming of cattle or sheep | Acute | 1-2 | Antimicrobial | + | - | + | 1:50 | | - |
|  | Female | 51 | Farmer | Exposure and farming of cattle or sheep | Acute | 0 | Untreated | + | + | + | 1:200 | | - |
|  | Female | 51 | Small business owner | Exposure and farming of cattle or sheep | Acute | 1-2 | Antimicrobial | + | + | + | 1:400 | | - |
|  | Male | 51 | Stockman | Exposure and farming of cattle or sheep | Acute | 0 | Untreated | + | + | + | 1:200 | | - |
|  | Male | 51 | Veterinarian | Exposure and farming of cattle or sheep | Acute | 1-2 | Antimicrobial | + | + | + | 1:200 | | - |
|  | Male | 51 | Veterinarian | Exposure and farming of cattle or sheep | Chronic | 1-2 | Immune enhancing agents | + | + | + | 1:100 | | - |
|  | Male | 51 | Stockman | Exposure and farming of cattle or sheep | Chronic | 1-2 | Immune enhancing agents | + | + | + | 1:100 | | - |
|  | Female | 51 | Farmer | Exposure and farming of cattle or sheep | Chronic | ≥3 | Immune enhancing agents | + | - | + | — | | - |
|  | Male | 51 | Veterinarian | Exposure and farming of cattle or sheep | Chronic | 1-2 | Immune enhancing agents | - | - | + | — | | - |
|  | Male | 51 | Farmer | Exposure and farming of cattle or sheep | Chronic | ≥3 | Immune enhancing agents | - | - | + | — | | - |
|  | Female | 51 | Stockman | Consumption history of raw meat or dairy | Chronic | ≥3 | Immune enhancing agents | + | - | + | 1:50 | | - |
|  | Male | 52 | Farmer | Exposure and farming of cattle or sheep | Acute | ≥3 | Immune enhancing agents | - | + | + | 1:800 | | - |
|  | Male | 52 | Veterinarian | Exposure and farming of cattle or sheep | Acute | ≥3 | Immune enhancing agents | - | + | + | 1:800 | | - |
|  | Female | 52 | Stockman | Consumption history of raw meat or dairy | Chronic | 0 | Untreated | + | + | + | 1:100 | | - |
|  | Male | 52 | Stockman | Exposure and farming of cattle or sheep | Chronic | ≥3 | Immune enhancing agents | + | - | + | 1:50 | | - |
|  | Female | 52 | Farmer | Exposure and farming of cattle or sheep | Chronic | ≥3 | Immune enhancing agents | + | - | + | 1:50 | | - |
|  | Male | 53 | Stockman | Exposure and farming of cattle or sheep | Acute | 1-2 | Immune enhancing agents | + | + | + | 1:200 | | - |
|  | Male | 53 | Farmer | Consumption history of raw meat or dairy | Acute | 0 | Untreated | + | - | + | 1:200 | | - |
|  | Male | 54 | Farmer | Exposure and farming of cattle or sheep | Acute | ≥3 | Unknown | + | + | + | 1:100 | | - |
|  | Male | 54 | Small business owner | Exposure and farming of cattle or sheep | Chronic | ≥3 | Immune enhancing agents | + | - | + | — | | - |
|  | Male | 54 | Veterinarian | Exposure and farming of cattle or sheep | Chronic | 0 | Untreated | - | - | + | — | | - |
|  | Female | 55 | Farmer | Exposure and farming of cattle or sheep | Acute | ≥3 | Immune enhancing agents | + | + | + | 1:200 | | - |
|  | Male | 55 | Stockman | Exposure and farming of cattle or sheep | Chronic | ≥3 | Immune enhancing agents | + | - | + | — | | - |
|  | Male | 55 | Farmer | Exposure and farming of cattle or sheep | Chronic | 1-2 | Unknown | + | - | + | 1:50 | | - |
|  | Female | 55 | Farmer | Exposure and farming of cattle or sheep | Chronic | ≥3 | Immune enhancing agents | - | - | + | — | | - |
|  | Male | 55 | Farmer | Exposure and farming of cattle or sheep | Acute | 0 | Untreated | + | - | + | 1:50 | | - |
|  | Male | 56 | Farmer | Exposure and farming of cattle or sheep | Acute | ≥3 | Immune enhancing agents | + | + | + | 1:100 | | - |
|  | Female | 57 | Farmer | Exposure and farming of cattle or sheep | Acute | 1-2 | Antimicrobial | + | + | + | 1:800 | | - |
|  | Male | 58 | Farmer | Exposure and farming of cattle or sheep | Chronic | ≥3 | Immune enhancing agents | + | + | + | 1:200 | | - |
|  | Male | 58 | Farmer | Exposure and farming of cattle or sheep | Chronic | ≥3 | Immune enhancing agents | + | + | + | 1:200 | | - |
|  | Female | 59 | Farmer | Exposure and farming of cattle or sheep | Acute | 1-2 | Antimicrobial | + | + | + | 1:400 | | - |
|  | Female | 59 | Farmer | Exposure and farming of cattle or sheep | Acute | 1-2 | Antimicrobial | + | + | + | 1:400 | | - |
|  | Male | 59 | Farmer | Unknown | Acute | 0 | Untreated | + | + | + | 1:100 | | - |
|  | Female | 59 | Farmer | Exposure and farming of cattle or sheep | Acute | 1-2 | Antimicrobial | + | + | - | 1:400 | | - |
|  | Male | 60 | Farmer | Exposure and farming of cattle or sheep | Chronic | 0 | Untreated | + | + | + | 1:100 | | - |
|  | Male | 60 | Veterinarian | Exposure and farming of cattle or sheep | Chronic | ≥3 | Immune enhancing agents | + | - | + | — | | - |
|  | Female | 61 | Farmer | Exposure and farming of cattle or sheep | Acute | 0 | Untreated | + | + | + | 1:100 | | - |
|  | Female | 61 | Small business owner | Exposure and farming of cattle or sheep | Acute | ≥3 | Antimicrobial | + | + | + | 1:200 | | - |
|  | Male | 61 | Farmer | Exposure and farming of cattle or sheep | Chronic | 1-2 | Antimicrobial | + | + | + | 1:800 | | - |
|  | Male | 62 | Stockman | Exposure and farming of cattle or sheep | Acute | 1-2 | Antimicrobial | - | + | + | 1:200 | | - |
|  | Male | 62 | Veterinarian | Exposure and farming of cattle or sheep | Chronic | ≥3 | Immune enhancing agents | - | - | + | 1:50 | | - |
|  | Female | 64 | Farmer | Exposure and farming of cattle or sheep | Chronic | ≥3 | Unknown | + | + | + | 1:400 | | - |
|  | Female | 67 | Farmer | Exposure and farming of cattle or sheep | Chronic | ≥3 | Unknown | - | - | + | — | | - |
|  | Female | 70 | Farmer | Exposure and farming of cattle or sheep | Acute | 1-2 | Antimicrobial | + | + | + | 1:100 | | - |
|  | Female | 70 | Farmer | Exposure and farming of cattle or sheep | Acute | 1-2 | Antimicrobial | + | + | + | 1:200 | | + |
|  | Female | 72 | Breeder | Exposure and farming of cattle or sheep | Chronic | ≥3 | Immune enhancing agents | - | - | + | — | | - |
|  | Male | 73 | Farmer | Unknown | Chronic | ≥3 | Unknown | + | + | + | 1:200 | | - |

Δ Patient is stratified into two clinical groups: acute phase (with symptoms for less than 6 months) and chronic phase (with symptoms for more than 6 months).

* Therapy course for brucellosis patients in hospital included multiple standard 20 days courses of treatment separated by an interval of 10 days. The number indicates the first two treatment courses with 1-2, the third course with 3, and 0 means untreated before. Disease durations are presented in day (d), month (m) or year (y).

# In the treatment regimen, Antimicrobial indicated the standard antibiotic treatment; Immune enhancing agents indicated the standard antibiotic treatment additionally receiving immune enhancing drugs.
